# The Diagnostic and Prognostic Utility of Transabdominal Ultrasound in Crohn's Disease: A Clinical Study

**DOI:** 10.1111/1751-2980.70009

**Published:** 2025-09-17

**Authors:** Chen Xie, Xin Wang, Jie Gu, Xue Qin Pang, Qin Hua Xi, Lan Xiang Zhu, Jun Sun, Wei Chang Chen

**Affiliations:** ^1^ Department of Gastroenterology The First Affiliated Hospital of Soochow University Suzhou Jiangsu Province China; ^2^ Department of Geriatric Medicine Suzhou Municipal Hospital Suzhou Jiangsu Province China; ^3^ Department of Ultrasound The First Affiliated Hospital of Soochow University Suzhou Jiangsu Province China

**Keywords:** bowel wall thickness, Crohn disease, Limberg score, prognosis, transabdominal ultrasound

## Abstract

**Objectives:**

In this retrospective study we aimed to assess the diagnostic, monitoring, and prognostic utility of transabdominal ultrasound (TBUS) in patients with Crohn's disease in China and evaluate the utility of 16‐week bowel wall thickness (BWT) reduction as a predictor of long‐term outcomes.

**Methods:**

Patients with CD, either newly or previously diagnosed, who received biologic therapy for the first time and underwent baseline TBUS and endoscopy between June 2022 and September 2023 were included, with follow‐up TBUS performed at Weeks 16 and 52 after the initiation of biologic therapy; clinical, ultrasound, laboratory, and disease activity data were collected.

**Results:**

Among the 60 patients, TBUS identified bowel wall thickening in 55 patients, with an average thickness of 7.36 ± 2.56 mm. The Limberg score of vascularization in the affected segments was ≥ 3 in 58.3% of the patients. Ascites, lymphadenopathy, and mesenteric fat hypertrophy were observed in 23.3%, 41.7%, and 41.7% of the patients, respectively. Significant correlations were found between baseline SES‐CD or CDAI and BWT (*r* = 0.650 for SES‐CD and 0.331 for CDAI) and the Limberg score (*r* = 0.538 for SES‐CD and 0.387 for CDAI). The receiver operating characteristic (ROC) curve analysis revealed high diagnostic accuracy for BWT (area under the ROC curve [AUROC] 0.973) and the Limberg score (AUROC 0.927). Follow‐up TBUS at Weeks 16 and 52 showed significant reductions in BWT and Limberg score. BWT reduction at Week 16 was significantly associated with CD clinical remission at Week 52 (*p* < 0.05).

**Conclusion:**

TBUS, particularly BWT and Limberg score, may serve as a useful noninvasive tool for diagnosis, monitoring, and prognosis in CD.

## Graphical abstract

Our study demonstrates the diagnostic and prognostic utility of transabdominal ultrasound (TBUS) in patients with Crohn's disease (CD). The key findings include significant correlations between bowel wall thickness (BWT), and disease activity indices such as the Simple Endoscopic Score for Crohn's Disease (SES‐CD), Crohn's Disease Activity Index (CDAI), and inflammatory biomarkers such as erythrocyte sedimentation rate (ESR), and C‐reactive protein (CRP), and fecal calprotectin (FC), as well as vascularization (Limberg score) with SES‐CD, CDAI, and ESR. Notably, reduction in BWT at Week 16 after the initiation of biologic therapy was significantly correlated with clinical remission of CD at Week 52, emphasizing the potential of TBUS as a dynamic monitoring tool for therapeutic response in CD.

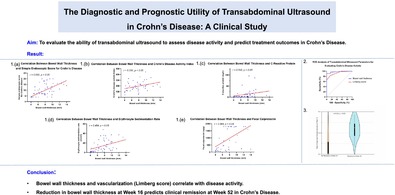

## 1 Introduction

Crohn's disease (CD) is a chronic, relapsing inflammatory disorder that may primarily affect any part of the gastrointestinal (GI) tract, with potential extraintestinal manifestations and immune dysregulation [1]. It is characterized by transmural inflammation and can be categorized into three phenotypes based on their disease behaviors, namely, non‐stricturing/non‐penetrating, stricturing, and penetrating. In China, the incidence of CD has been increasing during the recent years [2], with a peak age of disease onset between 30 and 34 years [3].

At present, a definitive gold standard for the diagnosis of CD is lacking worldwide, which requires a comprehensive approach including clinical evaluation, laboratory tests, imaging studies, endoscopy, and histopathological assessment [4]. Endoscopy remains the main diagnostic tool for CD diagnosis; however, it has certain limitations. For example, endoscopic examination focuses on the mucosal layer of the GI lumen and has potential difficulties in detecting penetrating complications such as fistula and abscess. In addition, inflammatory markers that are widely used in clinical practice, such as erythrocyte sedimentation rate (ESR), C‐reactive protein (CRP), and fecal calprotectin (FC), can be used for monitoring disease activity; however, as their sensitivity and specificity are suboptimal to those of endoscopy, whether they can be used to replace endoscopy needs to be verified [5].

Though transmural healing (TH) is not yet established as a formal therapeutic target in CD, it has been increasingly recognized as an adjunctive indicator to endoscopic remission, representing a deeper level of healing that correlates with improved patient outcomes. Accordingly, imaging modalities such as ultrasound, computed tomography enterography (CTE), and magnetic resonance enterography (MRE) play an important role in CD assessment [6]. Among these, transabdominal ultrasound (TBUS) has been reported to be an ideal modality for the monitoring of disease activity and complications in CD [7, 8]. Given its noninvasive nature, diagnostic accuracy, and cost‐effectiveness, TBUS can be particularly indicated for repeated evaluations during the chronic and relapsing–remitting episodes of the disease.

Previous studies have demonstrated that TBUS correlates well with disease activity and can predict treatment response [7, 8] and is a valuable tool for ongoing monitoring and therapeutic adjustments. The ECCO‐ESGAR guideline recommends TBUS, MRE, and/or capsule endoscopy as the first‐line options for assessing small bowel involvement in newly diagnosed CD [9]. However, existing research has largely focused on cross‐sectional associations or Western cohorts, with limited data on the prognostic utility of TBUS, especially in Asian populations.

Therefore, we conducted this study including 60 patients with CD in China to evaluate the diagnostic and monitoring utility of TBUS at baseline, Week 16, and Week 52, in comparison with endoscopic and laboratory markers, to assess the treatment‐related ultrasound changes over time, and to explore the prognostic value of early ultrasound response—which is defined as the rate of bowel wall thickness (BWT) reduction at Week 16—in predicting long‐term clinical outcomes.

## 2 Patients and Methods

### 2.1 Study Population

Patients with a confirmed diagnosis of CD who received biologic treatment for the first time at the Department of Gastroenterology, The First Affiliated Hospital of Soochow University (Suzhou, Jiangsu Province, China) between June 2022 and September 2023 were retrospectively enrolled. Diagnosis of CD was established based on a comprehensive evaluation of clinical manifestations, laboratory test results, imaging and endoscopic (colonoscopy and enteroscopy) findings, histopathological assessment, and follow‐up observations, in accordance with the *2023 Chinese national clinical practice guideline on diagnosis and management of Crohn's disease* [10]. The inclusion criteria were: (i) patients with previously or newly diagnosed CD who received biologic therapy for the first time during the study period (biologic‐naïve at enrollment); (ii) sufficient baseline TBUS and endoscopic data; and (iii) complete clinical and laboratory information for evaluation. There were no restrictions on patient's age, disease activity, or disease location/extent at enrollment. Follow‐up TBUS was performed at Weeks 16 and 52 according to the clinical indications and feasibility; thus, the number of patients included for analysis varied across different time points. Exclusion criteria included pregnancy during the study period, inability to undergo baseline TBUS or endoscopy, or insufficient baseline clinical data. None of the patients had undergone bowel resection or ileostomy. This retrospective study was approved by the Ethics Committee of the First Affiliated Hospital of Soochow University (no. 2024029), and written informed consent was waived due to the study design.

### 2.2 Data Collection

#### 2.2.1 Demographics, Clinical Manifestations, and Laboratory Test Indicators

Digital medical records of the patients were reviewed, and their demographics (age and gender), symptoms and signs, previous history of medication, and laboratory test indicators including ESR, CRP, and FC were extracted from the electronic medical record system of the hospital.

#### 2.2.2 Treatments and Assessment of Disease Activity

Biologics used for treating CD included ustekinumab (intravenous administration for induction at 6 mg/kg, then subcutaneous administration at 90 mg every 8 to 12 weeks), infliximab (intravenous administration at 5 mg/kg at baseline, Week 2, and Week 6, and every 8 weeks thereafter), and adalimumab (subcutaneous administration at 160 mg at baseline, 80 mg at Week 2, and 40 mg every 2 weeks thereafter).

Endoscopic disease activity at baseline was assessed using the Simple Endoscopic Score for Crohn's Disease (SES‐CD) [11], which evaluates four key endoscopic features—ulcer size, proportion of ulcerated surface, extent of affected mucosa, and presence of stenosis—across five intestinal segments, namely, the rectum, descending and sigmoid colon, transverse colon, ascending colon, and terminal ileum. Each endoscopic feature is graded on a scale of 0–3 points, with a maximum of 15 points, except for stenosis (range 0–11 points); the cumulative score ranges from 0 to 56 points. Based on the SES‐CD, disease activity of CD was categorized as follows: remission, ≤ 2; mild activity, 3–6; moderate activity, 7–15; and severe activity, ≥ 16.

The Montreal classification [12] was applied, including age at diagnosis (A1–A3), and disease location (L1–L4) and behavior (B1–B3 with perianal modifier) assessed at baseline.

In addition, the Crohn's Disease Activity Index (CDAI) [13] was applied to assess clinical disease activity at baseline, Week 16, and Week 52. Based on the CDAI score, CD activity was classified as follows: remission, < 150; mild activity, 150–220; moderate activity, 221–450; and severe activity, > 450.

Inflammatory markers, including ESR, CRP, and FC, were measured at baseline and at follow‐up visits when available.

#### 2.2.3 TBUS and Endoscopic Findings

We retrospectively reviewed the data of patients who had undergone both baseline TBUS and endoscopic examinations within 30 days prior to the initiation of biologic therapy, with the interval between the two procedures not exceeding 1 month. Follow‐up TBUS assessments at Weeks 16 and 52, when available, were also reviewed, and the findings were systematically recorded.

### 2.3 TBUS Examination

All TBUS procedures were performed using the GE LOGIQ E9 Ultrasound Machine (GE Healthcare Technologies, Madison, Wisconsin, USA) by one single sonographer who had performed TBUS examinations for over 10 years. Prior to the TBUS examination, the patient was required to fast for at least 8 h. During the procedure, the patient was placed in the supine position, with the abdomen fully exposed. The examination was initiated with a convex probe (C1‐6) from the right iliac fossa, moving in a clockwise manner to assess the terminal ileum, cecum, and colonic segments, followed by the evaluation of the small bowel according to the Cole classification used in GI radiology: Group 1, duodenum; Group 2, proximal jejunum in the left upper abdomen; Group 3, distal jejunum in the left mid‐abdomen; Group 4, proximal ileum in the right mid‐abdomen; Group 5, mid ileum in the right lower‐mid abdomen; and Group 6, distal ileum in the pelvis and right lower abdomen. Intestinal segments with any suspicious lesions were further examined with a linear probe (9L‐D) to assess the extent of BWT, mesenteric fat hypertrophy, lymphadenopathy, presence of stenosis, prestenotic dilatation, fistulas, or abscesses, as described previously [14]. Abdominal effusion was also evaluated. The color Doppler flow imaging (CDFI) was used to assess vascularity within the bowel wall. Disease activity and complications were also recorded based on the most severely affected intestinal segment.

The following parameters were recorded and analyzed:

- BWT was measured using a linear probe at the thickest point of the affected bowel segment, defined as the vertical distance from the gas interface in the lumen to the serosal layer [14]. Measurements were reported in millimeters.
- Limberg score was calculated with CDFI to assess the intra‐ and extramural blood flow. The Limberg's semiquantitative grading system was used, ranging from 0 (normal) to 4 (marked vascularity extending to mesenteric vessels), with higher scores indicating a more severe activity [15].
- Mesenteric fat hypertrophy was defined as a hyperechoic tissue area or “mass effect wrapping” adjacent to the diseased bowel wall with a maximum thickness of ≥ 5 mm [14, 16], which was recorded as either present or absent.
- Lymphadenopathy was defined as inflammatory mesenteric lymph nodes related to CD, presenting as oval or elongated hypoechoic nodes of over 5 mm in diameter [14], which was recorded as either present or absent.
- Fistula was defined as hypoechoic tracts or areas between ileal loops, or hypoechoic peri‐intestinal tracts or areas connecting to adjacent organs or the skin, with significant bowel wall thickening with or without air bubbles or feces [14], which was recorded as either present or absent.
- Abscess was identified as an irregular, non‐space‐occupying hypo‐anechoic lesion containing fluid and gaseous artifacts, with posterior enhancement, of over 2 cm in size [14, 17, 18], which was recorded as either present or absent.
- Bowel lumen stenosis was defined as bowel wall thickening with lumen narrowing (< 1 cm in diameter) and prestenotic dilatation (> 25 mm) [19], which was recorded as present or absent.
- Ascites was defined as an anechoic fluid collection in the peritoneal cavity or between the intestinal loops [19], which was recorded as present or absent.

### 2.4 Statistical Analysis

All the statistical analyses were performed by using the SPSS Statistics version 27.0 (IBM, Armonk, NY, USA). Categorical variables were presented as numbers and percentages or frequencies. Continuous variables were tested for normality using the Shapiro–Wilk test and inspection of Q–Q plots. Normally distributed continuous variables were expressed as mean ± standard deviation, whereas non‐normally distributed variables were reported as median and interquartile range (IQR). The Spearman's rank correlation analysis was utilized to evaluate the associations between continuous and ordinal variables, and point‐biserial correlation was used for associations between continuous and binary variables. For independent subgroup comparisons, the Mann–Whitney *U*‐test was applied, while the Wilcoxon signed‐rank test was used for paired comparisons of pre‐ and posttreatment samples. Differences in categorical variables were analyzed by using the Chi‐square test. Diagnostic performance of TBUS was evaluated using the receiver operating characteristic (ROC) curve analysis, and the area under the ROC curve (AUROC) was calculated, together with the optimal thresholds, sensitivity, specificity, and the Youden's index (calculated as sensitivity plus specificity minus 1). A *p* value of less than 0.05 was considered statistically significant.

## 3 Results

### 3.1 Baseline Characteristics of the Patients

A total of 75 patients with CD underwent TBUS at the First Affiliated Hospital of Soochow University between June 2022 and September 2023. Of them, 15 patients were excluded because of incomplete TBUS or endoscopy data, or an interval exceeding 1 month between TBUS and endoscopic examination. Thus, 60 patients were included in the final analysis, comprising those with newly and previously diagnosed CD who initiated biologic therapy during the study period.

The mean age of these 60 patients was 34.9 ± 13.0 years (range 15–66 years), and there was a male predominance (78.3%, *n* = 47). Regarding the inflammatory markers, the median levels of CRP, FC, and ESR were 4.5 mg/L (IQR 13.4 mg/L), 794.9 μg/g (IQR 1619.8 μg/g), and 8.0 mm/h (IQR 22 mm/h), respectively.

The median CDAI and SES‐CD of the patients were 190.4 (IQR 106.1) and 7.5 (IQR 10), respectively. According to the Montreal classification [12], most patients presented with ileal (L1) disease (61.7%, *n* = 37), followed by ileocolonic (L3) disease in 14 (23.3%) patients, and colonic (L2) disease in 9 (15.0%) patients. In addition, 60.0% of the patients presented with a non‐stricturing, non‐penetrating disease behavior (B1), while the other 40.0% of patients had a stricturing (B2) disease behavior. None of them had penetrating disease (B3). Regarding age at diagnosis, 3 (5.0%) patients were classified as A1 (< 17 years), 40 (66.7%) as A2 (17–40 years), and 17 (28.3%) as A3 (> 40 years), respectively. No perforation, fistula, or abscess was observed.

### 3.2 TBUS Findings

All patients underwent TBUS examination at baseline; among them, 55 (91.7%) exhibited varying degrees of bowel wall thickening, with a mean BWT of 7.36 ± 2.56 mm (range 4–14 mm). The Limberg scores 0 to 4 for evaluating the vascularization of the affected segments were observed in 3 (5.0%), 12 (20.0%), 10 (16.7%), 25 (41.7%), and 10 (16.7%) cases, respectively. In addition, ascites were observed in 14 (23.3%) patients, while mesenteric lymphadenopathy and mesenteric fat hypertrophy were found in 25 (41.7%) patients each. Follow‐up TBUS examinations were performed in 16 patients at Weeks 16 and 52 (data shown below).

### 3.3 Correlation Between Baseline TBUS Parameters and Baseline CD Disease Activity

TBUS parameters at baseline were analyzed for their correlations with baseline SES‐CD, CDAI, and levels of inflammatory markers (ESR, CRP, and FC).

#### 3.3.1 BWT

**FIGURE 1:**
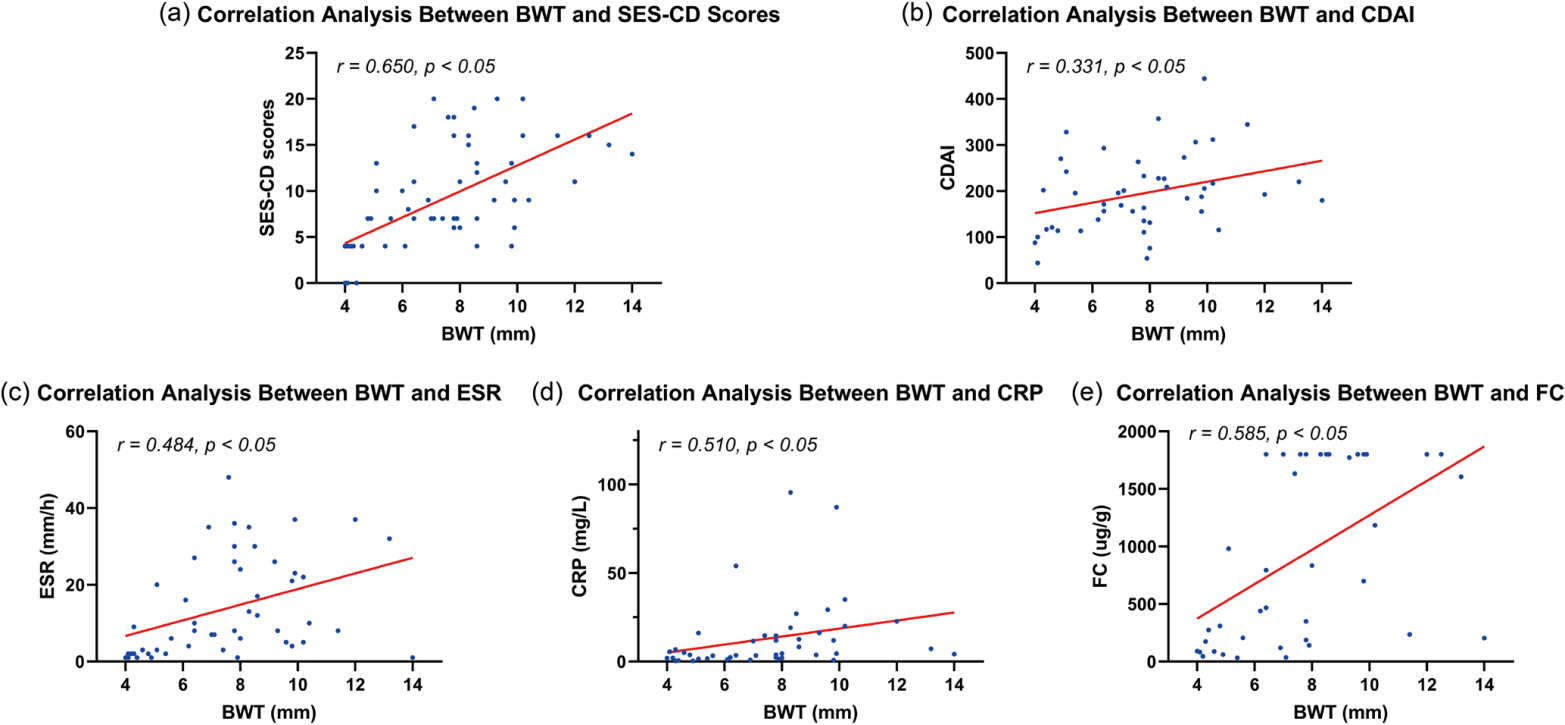
Correlation analysis between baseline bowel wall thickness (BWT) and (a) Simple Endoscopic Score for Crohn's Disease (SES‐CD), (b) Crohn's Disease Activity Index (CDAI), (c) erythrocyte sedimentation rate (ESR), (d) C‐reactive protein (CRP), and (e) fecal calprotectin (FC) at baseline.

The Spearman's correlation analysis revealed significant positive correlations between BWT and SES‐CD score (*r* = 0.650), CDAI scores (*r* = 0.331), ESR (*r* = 0.484), CRP (*r* = 0.510), and FC (*r* = 0.585) (all *p* < 0.05) (Figure 1, Table 1).

**TABLE 1:** Correlation between baseline ransabdominal ultrasound (TBUS) parameters and Simple Endoscopic Score for Crohn's Disease (SES‐CD), Crohn's Disease Activity Index (CDAI), and clinical inflammatory markers at baseline.

Parameters	SES‐CD		CDAI		ESR		CRP		FC	
	*r*	*p* value	*r*	*p* value	*r*	*p* value	*r*	*p* value	*r*	*p* value
BWT	0.650	**< 0.05**	0.331	**< 0.05**	0.484	**< 0.05**	0.510	**< 0.05**	0.585	**< 0.05**
Limberg score	0.538	**< 0.05**	0.387	**< 0.05**	0.540	**< 0.05**	0.273	0.069	0.531	**< 0.05**
Mesenteric fat hypertrophy	0.321	**< 0.05**	0.242	0.105	0.204	0.148	0.241	0.099	0.099	0.518
Lymphadenopathy	0.264	**< 0.05**	0.242	0.106	0.132	0.351	0.326	**< 0.05**	0.131	0.389
Ascites	0.050	0.705	0.153	0.308	0.122	0.391	−0.069	0.643	0.219	0.148

*Note:* Bold characters indicate statistical significance.

Abbreviations: BWT, bowel wall thickness; CRP, C‐reactive protein; ESR, erythrocyte sedimentation rate; FC, fecal calprotectin.

#### 3.3.2 Limberg Score

**FIGURE 2:**
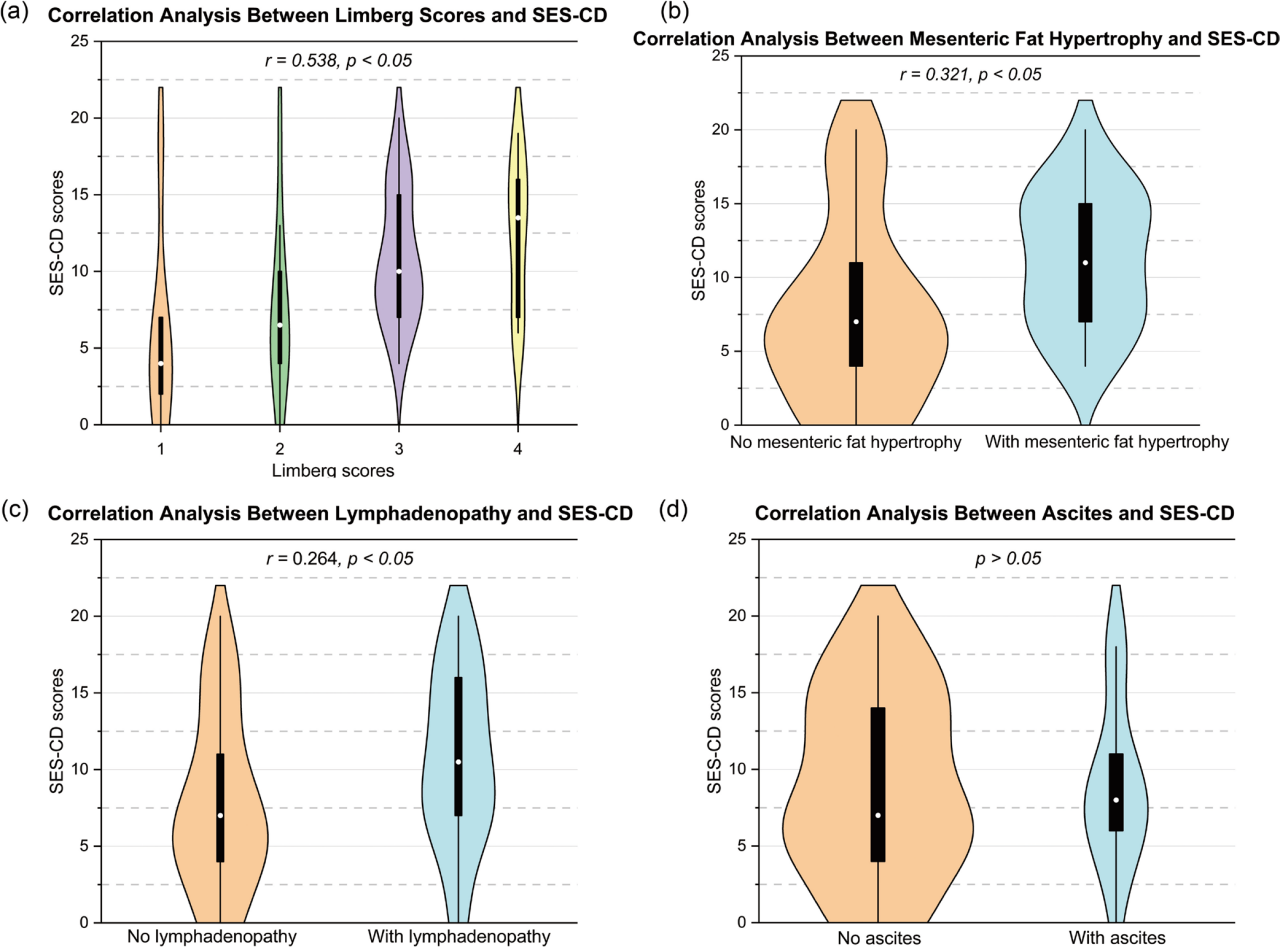
Correlation analysis between baseline Simple Endoscopic Score for Crohn's Disease (SES‐CD) and other transabdominal ultrasound (TBUS) parameters at baseline, including (a) Limberg score, (b) mesenteric fat hypertrophy, (c) lymphadenopathy, and (d) ascites.

**FIGURE 3:**
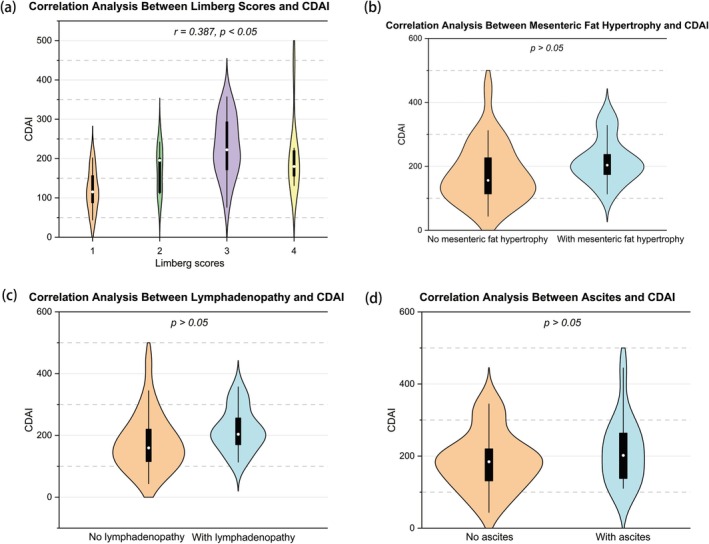
Correlation analysis between baseline Crohn's Disease Activity Index (CDAI) and other transabdominal ultrasound (TBUS) parameters at baseline, including (a) Limberg score, (b) mesenteric fat hypertrophy, (c) lymphadenopathy, and (d) ascites.

**FIGURE 4:**
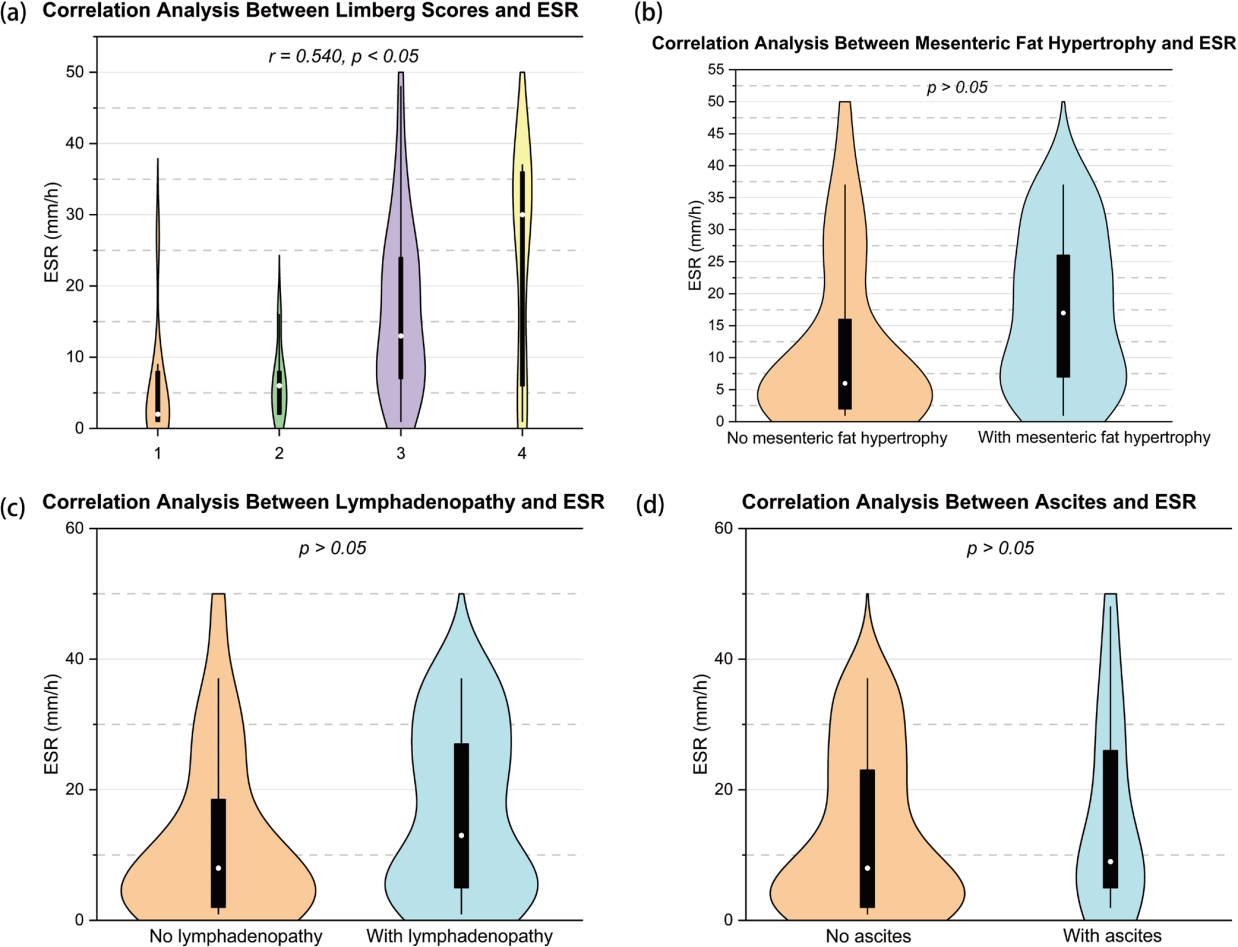
Correlation analysis between baseline erythrocyte sedimentation rate (ESR) and other transabdominal ultrasound (TBUS) parameters at baseline, including (a) Limberg score, (b) mesenteric fat hypertrophy, (c) lymphadenopathy, and (d) ascites.

**FIGURE 5:**
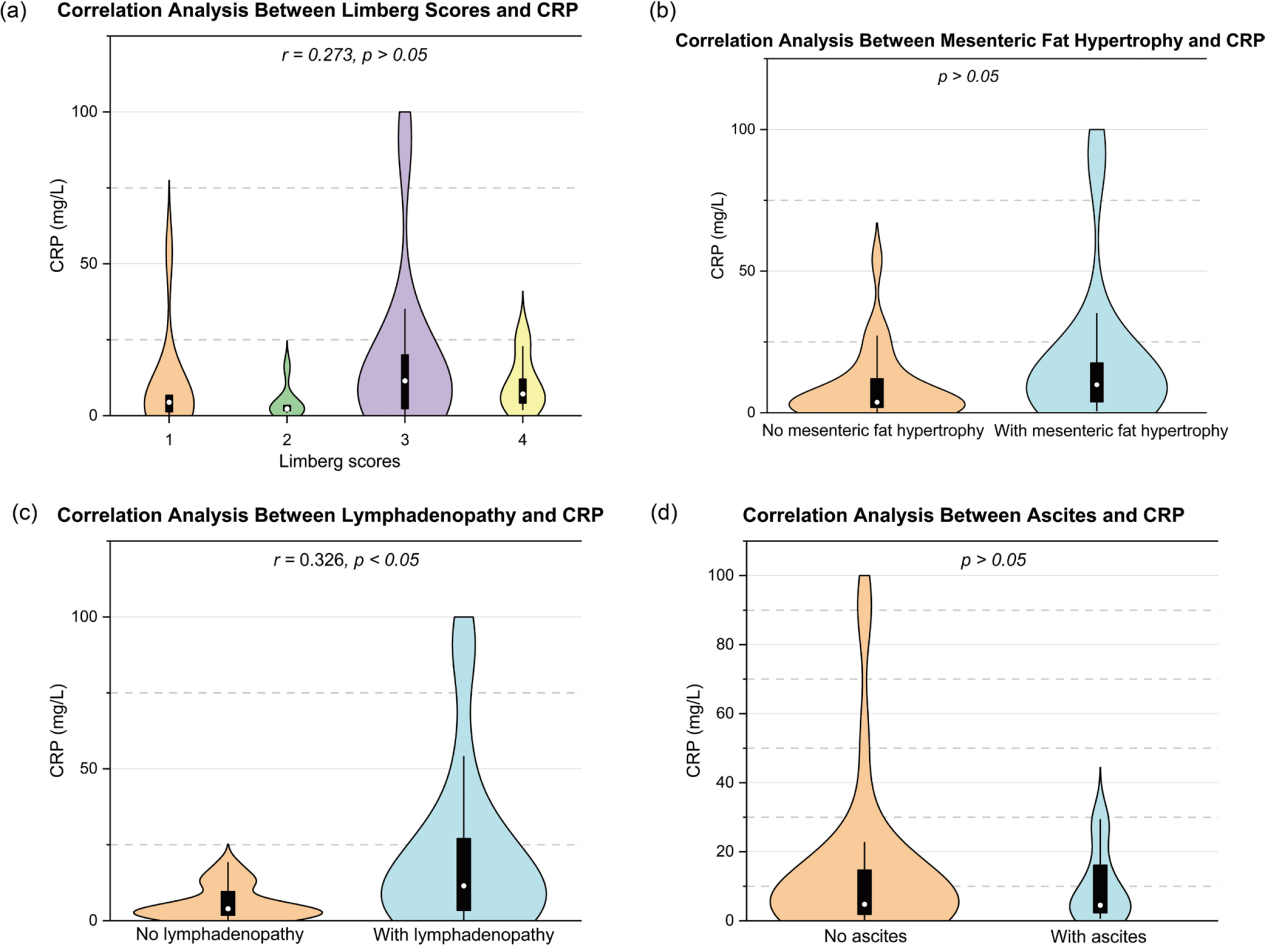
Correlation analysis between baseline C‐reactive protein (CRP) and other transabdominal ultrasound (TBUS) parameters at baseline, including (a) Limberg score, (b) mesenteric fat hypertrophy, (c) lymphadenopathy, and (d) ascites.

**FIGURE 6:**
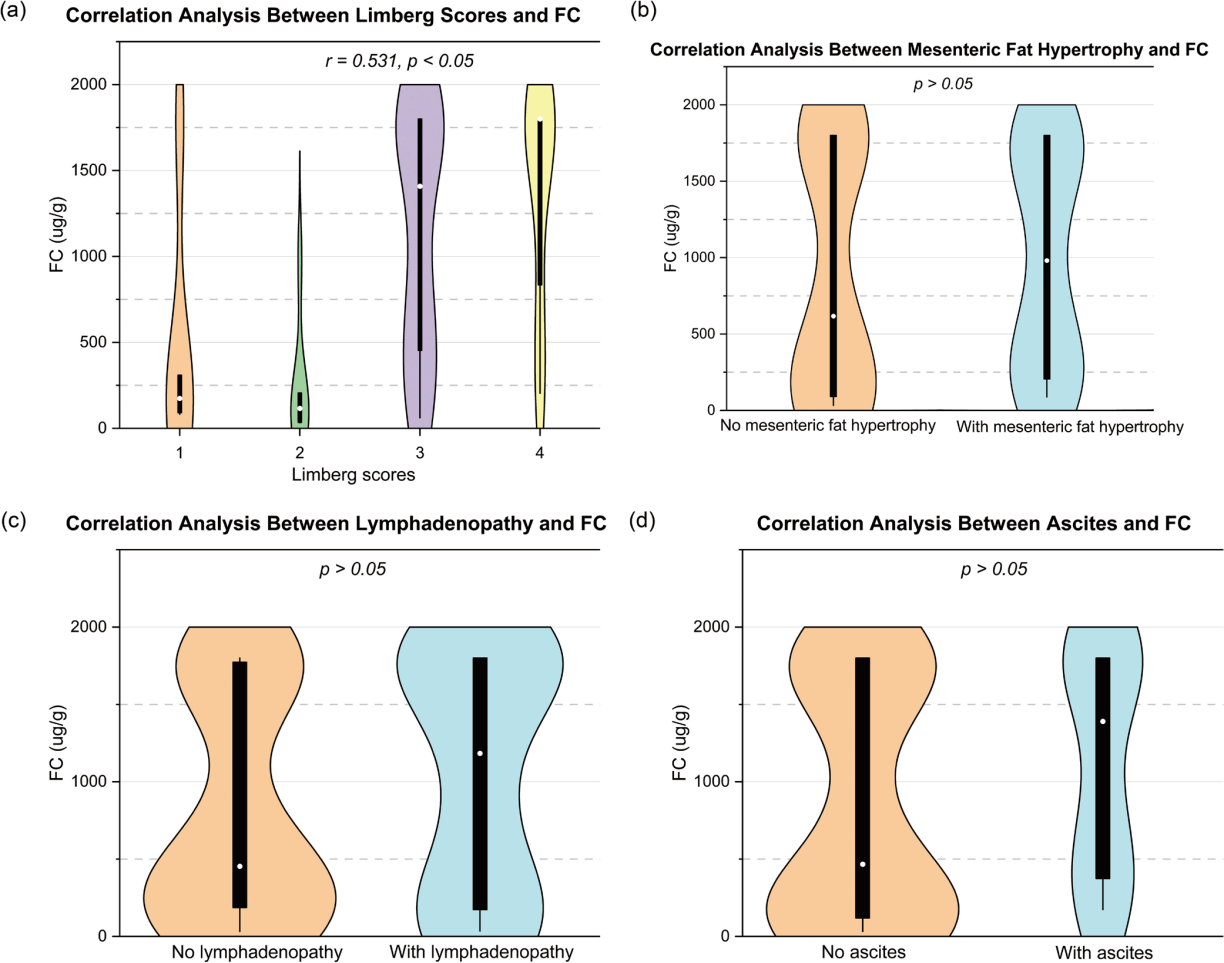
Correlation analysis between baseline fecal calprotectin (FC) and other transabdominal ultrasound (TBUS) parameters at baseline, including (a) Limberg score, (b) mesenteric fat hypertrophy, (c) lymphadenopathy, and (d) ascites.

The Limberg score was significantly correlated with SES‐CD (*r* = 0.538), CDAI (*r* = 0.387), ESR (*r* = 0.540), and FC (*r* = 0.531) (all *p* < 0.05), but not with CRP (*p* > 0.05) (Figures 2, 3, 4, 5, 6, Table 1).

#### 3.3.3 Other TBUS Parameters

Point‐biserial correlation analysis showed significant associations between SES‐CD and mesenteric fat hypertrophy (*r* = 0.321) and lymphadenopathy (*r* = 0.264) (both *p* < 0.05), while no significant correlations was observed between SES‐CD and ascites (*p* > 0.05) (Figures 2, Table 1).

Lymphadenopathy was found to be correlated with CRP (*r* = 0.326, *p* < 0.05; Figure 5), whereas mesenteric fat hypertrophy and ascites were not associated with CRP, ESR, or FC (all *p* > 0.05) (Figures 4, 5, 6, Table 1).

### 3.4 The ROC Analysis of Baseline TBUS Parameters for Evaluating CD Disease Activity

**FIGURE 7:**
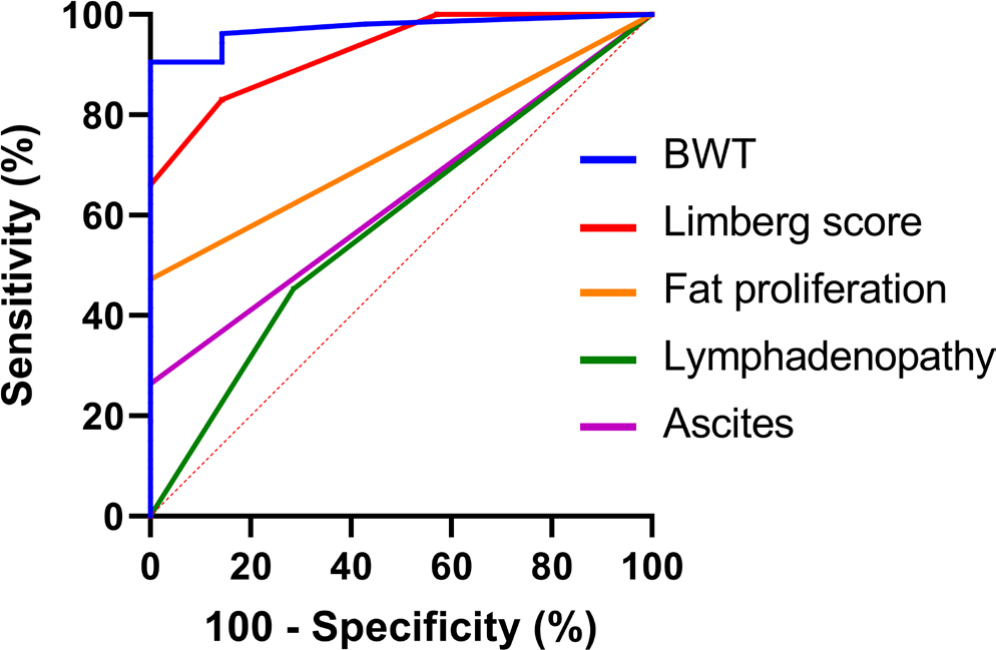
The receiver operating characteristic (ROC) analysis of transabdominal ultrasound (TBUS) parameters at baseline for diagnosing Crohn's disease (CD) activity. BWT, bowel wall thickness.

Using SES‐CD as the reference standard for endoscopic disease activity, patients were classified into active and remission groups. The ROC curves were then generated to evaluate the diagnostic performance of TBUS parameters (Figure 7), and their AUROCs are summarized in Table 2.

**TABLE 2:** The receiver operating characteristic (ROC) analysis of baseline transabdominal ultrasound (TBUS) parameters for diagnosing the activity of Crohn's disease.

TBUS parameters	AUROC	95% CI	Sensitivity (%)	Specificity (%)	Youden's index	*p* value
BWT	0.973	0.934–1.000	90.6	100	0.906	**< 0.05**
Limberg score	0.927	0.847–1.000	83.0	85.7	0.687	**< 0.05**
Mesenteric fat hypertrophy	0.736	0.590–0.881	47.2	100	0.472	**< 0.05**
Lymphadenopathy	0.584	0.365–0.802	45.3	71.4	0.167	0.475
Ascites	0.640	0.433–0.847	28.0	100	0.280	0.306

*Note:* Bold characters indicate statistical significance.

Abbreviations: AUROC, area under the ROC curve; BWT, bowel wall thickness; CI, confidence interval.

The ROC curve demonstrated an optimal cut‐off value of 4.5 mm for BWT, which achieved the highest Youden's index (0.906) with an AUROC of 0.973 (*p* < 0.05). The corresponding sensitivity and specificity for identifying active CD were 90.6% and 100%, respectively.

For the Limberg score, the optimal cut‐off value was 2, yielding a Youden's index of 0.687 and an AUROC of 0.927 (*p* < 0.05), and the sensitivity and specificity were 83.0% and 85.7%, respectively.

Mesenteric fat hypertrophy showed an AUROC of 0.736 (95% CI 0.590–0.881; *p* < 0.05), with sensitivity of 47.2% and specificity of 100%. In contrast, lymphadenopathy had a lower AUROC of 0.584 (95% CI 0.365–0.802; *p* = 0.475), with sensitivity and specificity of 45.3% and 71.4%. Similarly, ascites yielded an AUROC of 0.640 (95% CI 0.433–0.847; *p* = 0.360), with sensitivity and specificity of 28.0% and 100%, respectively.

### 3.5 TBUS for the Assessment of Treatment Response in CD Patients

Of the 60 patients, 16 underwent follow‐up TBUS assessments at both Weeks 16 and 52 after initiation of biologic therapy. Among them, 13 received ustekinumab, 2 received infliximab, and the remaining patient received adalimumab. Treatment regimens were maintained throughout the study period without adjustment.

#### 3.5.1 Changes in TBUS Parameters After Treatment

**FIGURE 8:**
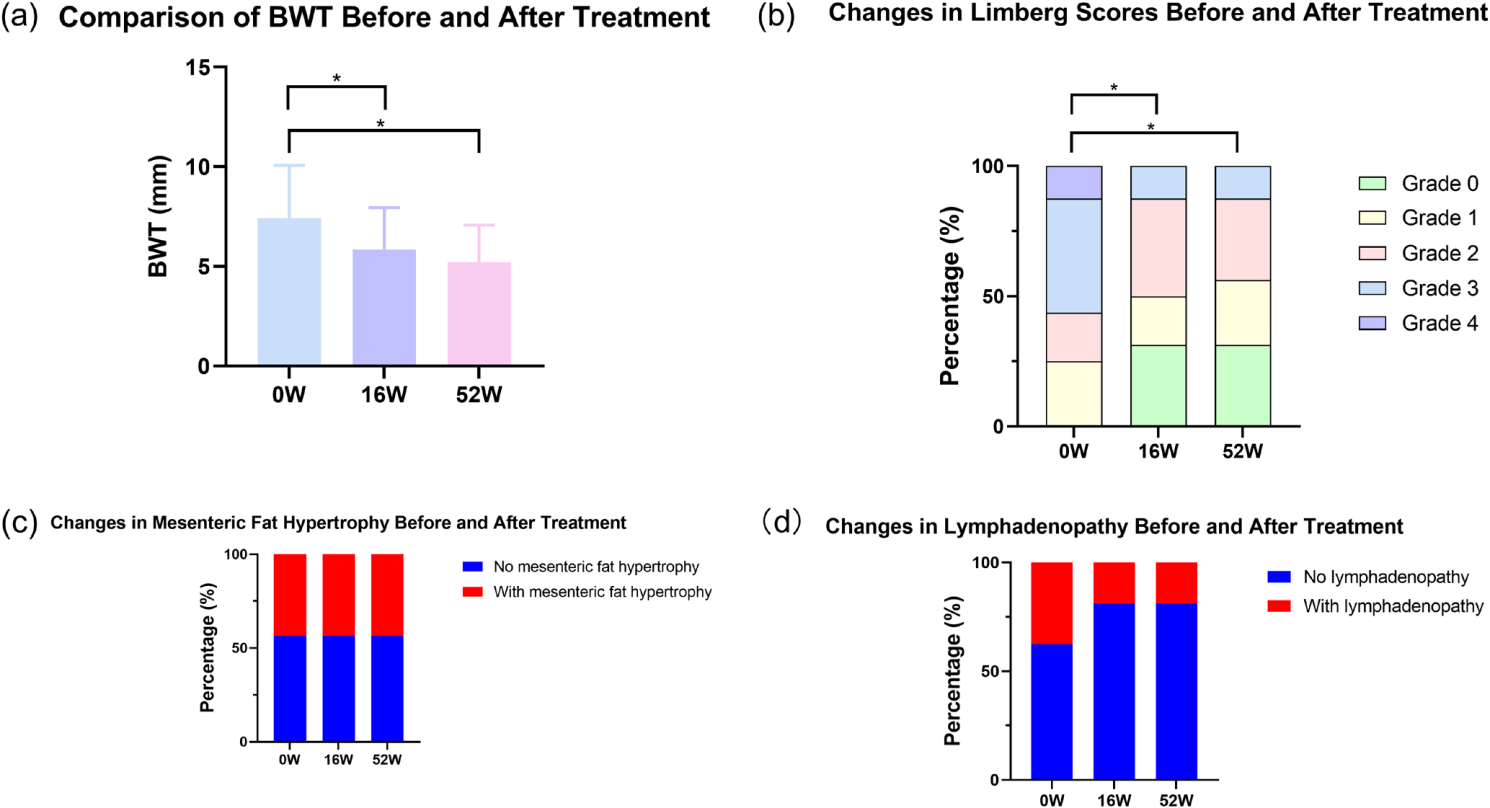
Changes in transabdominal ultrasound (TBUS) parameters including (A) bowel wall thickness (BWT), (B) Limberg score, (C) mesenteric fat hypertrophy, and (D) lymphadenopathy at baseline, at 16 and 52 weeks after the initiation of the treatment among the 16 patients underwent follow‐up TBUS examination. ^*^ *p* < 0.05.

At Week 16, the mean BWT of these 16 patients was 5.85 ± 2.11 mm, showing a significant reduction of 20.7% ± 11.8% from baseline (*p* < 0.05). In addition, the post‐treatment Limberg score was 0 in five (31.3%) patients, 1 in three (18.8%) patients, 2 in six (37.5%) patients, 3 in two (12.5%) patients, but 4 in none of the patients. The Wilcoxon signed‐rank test revealed a significant change in Limberg score compared to the baseline (*p* < 0.05). In addition, mesenteric fat hypertrophy and lymphadenopathy were found in seven (43.8%) and three (18.8%) patients, respectively, after treatment, though their changes from baseline were not statistically significant (both *p* > 0.05) (Figure 8).

At Week 52, the mean BWT was 5.22 ± 1.86 mm, showing a significant reduction of 27.4% ± 17.0% from baseline (*p* < 0.05). The Limberg scores 0 to 4 were found in five (31.3%), four (25.0%), five (31.3%), two (12.5%), and none of the patients, respectively. The Wilcoxon signed‐rank test indicated a significant change in Limberg score compared to the baseline (*p* < 0.05). In addition, mesenteric fat hypertrophy and lymphadenopathy were found in seven (43.8%) and three (18.8%) patients, respectively, with no significant change from the baseline (both *p* > 0.05) (Figure 8).

#### 3.5.2 Predictive Value of TBUS Parameters at Week 16 in Evaluating Treatment Response in CD

Using CDAI as the reference at Week 52, 14 (87.5%) patients achieved disease remission, whereas the other 2 (12.5%) patients had mildly active CD. Moderate or severe disease was not observed.

**FIGURE 9:**
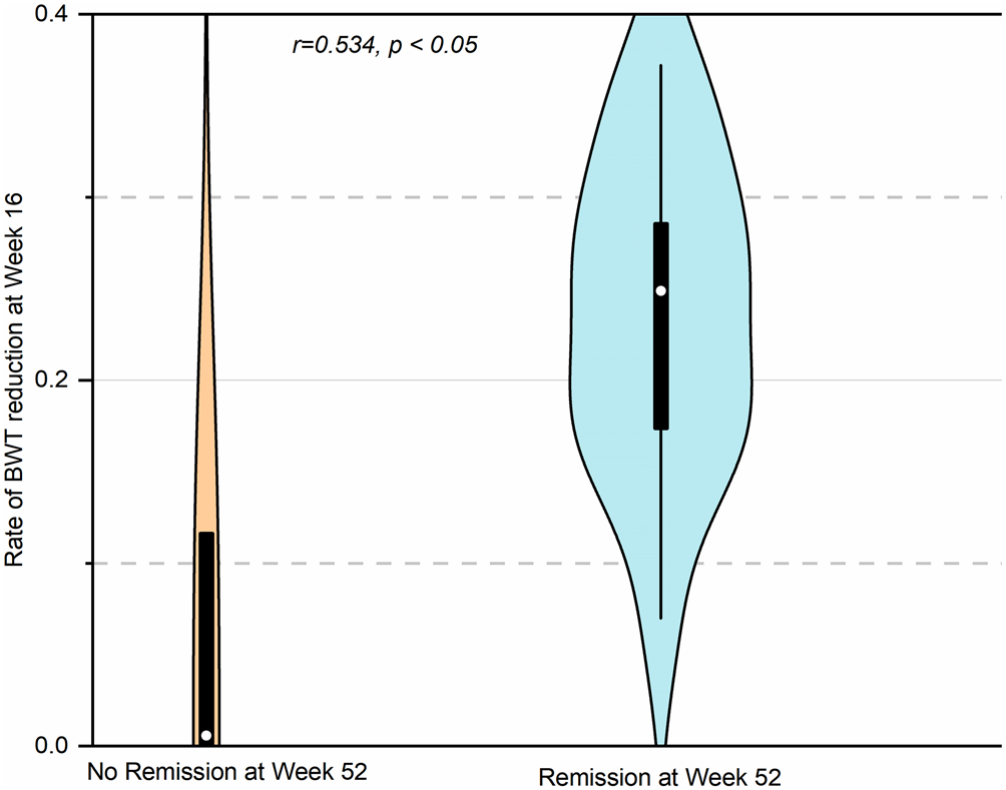
Correlation between the rate of bowel wall thickness (BWT) reduction at Week 16 and clinical remission of Crohn's disease (CD) at Week 52 after the initiation of the treatment.

A significant positive correlation was found between the rate of BWT reduction at Week 16 and clinical remission of CD at Week 52 (*r* = 0.534, *p* < 0.05) (Figure 9). While changes in Limberg score, mesenteric fat hypertrophy, or lymphadenopathy at Week 16 were not associated with clinical remission at Week 52 (*p* > 0.05).

## 4 Discussion

In the current study, we evaluated the diagnostic and prognostic utility of TBUS in patients with CD and found a significant correlation between BWT and indices for disease activity such as SES‐CD, CDAI, and inflammatory biomarkers (ESR, CRP, and FC), and between vascularization (Limberg score) and SES‐CD, CDAI, and ESR. Notably, reduction in BWT at Week 16 was significantly associated with clinical remission of CD at Week 52, emphasizing the potential of TBUS as a dynamic monitoring tool for treatment response.

We found that BWT was correlated with disease activity in CD, as shown by SES‐CD and CDAI, and had a good performance for evaluating disease activity, which is consistent with previous findings. Yaguchi et al. described an increase in BWT with progressive macroscopic disease severity in surgically resected specimens in CD patients [20]. Similarly, Goertz et al. demonstrated that BWT > 3 mm was a highly specific marker for histopathological inflammation of CD in the terminal or neoterminal ileum [15].

Notably, we demonstrated that early reductions in BWT at Week 16 after the initiation of treatment were correlated with clinical remission at Week 52. While Hoffmann et al. have revealed that a decrease in BWT of ≥ 1 mm correlates with short‐term response (at Week 8) to ustekinumab [21]. Our findings might extend this short‐term evidence to the prediction of long‐term outcome by linking early BWT change to long‐term clinical remission, offering a potentially novel application of TBUS in treatment monitoring.

Vascularization, as measured by the Limberg score, has been established as a reliable indicator of disease activity and inflammation [22]. Consistent with these studies, our results show strong correlations between the Limberg score and SES‐CD or CDAI. In addition, improvement in vascularization after treatment further supports its role in evaluating therapeutic response. These findings reinforce the value of vascularization as a supplementary parameter in TBUS evaluation.

While BWT and vascularization (Limberg score) were the primary ultrasound parameters of interest in our study, we also assessed mesenteric fat hypertrophy and lymphadenopathy in relation to disease activity. Despite its suboptimal sensitivity, mesenteric fat hypertrophy demonstrated moderate specificity for identifying active disease. These findings are consistent with prior studies, which have highlighted mesenteric fat hypertrophy and lymphadenopathy as supportive, albeit secondary, markers in CD evaluation [23, 24].

The diagnostic performance of TBUS for disease activity is comparable with that of MRE/CTE, as reported in previous studies [25, 26]. However, TBUS offers distinct advantages, including cost‐effectiveness, absence of radiation, and real‐time applicability. These advantages, in combination with the ability to monitor dynamic changes, reveal that TBUS may be a practical tool for long‐term CD monitoring.

Our study presents several methodological and clinical strengths. By focusing on a Chinese CD cohort, the current study contributes region‐specific evidence and addresses a limited availability of TBUS‐based research in Asian populations. Unlike previous cross‐sectional studies, the retrospective study design incorporated longitudinal follow‐up at Weeks 16 and 52, enabling the assessment of both short‐ and long‐term treatment outcomes. All patients underwent baseline TBUS prior to the initiation of biologic therapy, which minimized potential confounders. Notably, we demonstrated that early reductions in BWT at Week 16 might be used for the prediction of clinical remission at Week 52, highlighting the potential of TBUS as a dynamic, noninvasive tool for long‐term disease monitoring.

Nonetheless, there were some limitations to this study. First, this was a single‐center retrospective study with a small sample size, which limits the generalizability of our findings among different populations in varying regions. Second, while BWT and vascularization were confirmed as robust ultrasound parameters, other features such as mesenteric fat hypertrophy and lymphadenopathy were not fully evaluated, and we did not compare the performance of TBUS with other imaging modalities (e.g., MRE or CTE) to assess TH. Third, the total SES‐CD score was used to evaluate the endoscopic disease activity in CD, but the segmental scores were not assessed for their correlations. Fourth, clinical outcomes were assessed using CDAI, which—despite its widespread use in clinical practice—cannot directly reflect mucosal healing or TH. In the absence of routine follow‐up endoscopy or imaging in this retrospective cohort, CDAI was adopted as the primary long‐term indicator to ensure sufficient data collection. However, similar retrospective studies have also employed CDAI when objective end points were not universally available [27, 28], and the STRIDE‐II consensus [6] regards clinical remission (CDAI < 150) as the treatment goal, especially when interpreted alongside objective markers. In addition, a small subset of patients had achieved partial remission with conventional therapy or enteral nutrition before initiating biologics, which may have influenced baseline TBUS findings. Moreover, though all patients completed assessments at baseline, only 16 of them underwent TBUS at Weeks 16 and 52. Despite these limitations, this study provides important real‐world evidence supporting the role of TBUS in monitoring and predicting treatment response in CD. Future large‐scale prospective studies with standardized objective end points, such as mucosal healing, TH, and validated intestinal ultrasound scores, are warranted to verify our findings.

## 5 Conclusions

In this study, we demonstrated the diagnostic and prognostic potential of TBUS in CD, with BWT and the Limberg score serving as potentially reliable markers for the evaluation of disease activity and treatment response. Early BWT reduction may be used to predict long‐term clinical remission, showing that TBUS can be applied as a tool for noninvasive, dynamic monitoring in CD in clinical practice. Furthermore, this study provides region‐specific longitudinal evidence to the limited TBUS literature and supports the broader integration of TBUS into routine CD management.

## Conflicts of Interest

The authors declare no conflicts of interest.

## Data Availability

The data that support the findings of this study are available from the corresponding author upon reasonable request.
